# The effects of vitamin D supplementation on endothelial activation among patients with metabolic syndrome and related disorders: a systematic review and meta-analysis of randomized controlled trials

**DOI:** 10.1186/s12986-018-0320-9

**Published:** 2018-11-29

**Authors:** Reza Tabrizi, Maryam Akbari, Kamran B. Lankarani, Seyed Taghi Heydari, Fariba Kolahdooz, Zatollah Asemi

**Affiliations:** 1 Health Policy Research Center, Institute of Health, Student Research Committee, Shiraz University of Medical Sciences, Shiraz, Iran; 2 Health Policy Research Center, Institute of Health, Shiraz University of Medical Sciences, Shiraz, Iran; 3 Indigenous and Global Health Research, Department of Medicine, University of Alberta, Edmonton, Canada; 4 Research Center for Biochemistry and Nutrition in Metabolic Diseases, Kashan University of Medical Sciences, Kashan, IR Iran

**Keywords:** Vitamin D supplementation, Endothelial activation, Metabolic syndrome, Meta-analysis

## Abstract

**Background and objective:**

The current systematic review and meta-analysis of randomized controlled trials (RCTs) was conducted to summarize the effect of vitamin D supplementation on endothelial activation among patients with metabolic syndrome and related disorders.

**Methods:**

Cochrane library, Embase, PubMed, and Web of Science database were searched to identify related RCTs published before 30th April 2018. The heterogeneity among the included studies was assessed using Cochran’s Q test and I-square (I^2^) statistic. Data were pooled by using the random-effect model and standardized mean difference (SMD) was considered as summary effect size.

**Results:**

Fourteen clinical trials that contained a total of 1253 participants were included in the current meta-analysis. Vitamin D supplementation significantly decreased von willebrand factor (vWF) (SMD -0.27; 95% CI, − 0.46, − 0.08; *P* = 0.006; I^2^:40.5%). However, we found no significant impact of vitamin D supplementation on intercellular adhesion molecule 1(ICAM-1) (SMD -1.96; 95% CI, − 4.02, 0.09; *P* = 0.06; I^2^:97.4%), vascular celladhesion molecule 1 (VCAM-1) (SMD -0.50; 95% CI, − 1.19, 0.19; *P* = 0.15; I^2^:91.2%), on E-selectin (SMD -0.04; 95% CI, − 0.36, 0.28; *P* = 0.81; I^2^:78.8%) and endothelin (SMD -0.49; 95% CI, − 1.18, 0.19; P = 0.15; I^2^:90.5%). The pooled data from trials of vitamin D supplementation with dosage of ≤4000 IU/day (− 0.37, 95% CI: -0.65, − 0.10, I^2^: 73.5%) significantly reduced vWF concentrations, while there was no effect of vitamin D supplementation on vWF concentrations among trials with the dosage of intervention > 4000 IU/day (− 0.17, 95% CI: -0.43, 0.10, I^2^: 0.0%). VWF concentrations significantly reduced in pooled data from trials with duration study ≤8 weeks (− 0.37, 95% CI: -0.67, − 0.07, I^2^: 60.6%), but there was no effect of vitamin D supplementation on vWF concentrations among trials with > 8 weeks (− 0.20, 95% CI: -0.45, 0.05, I^2^: 0.0%). While there was no effect of vitamin D supplementation on vWF concentrations among trials with total sample size of ≤60 patients (− 0.03, 95% CI: -0.42, 0.36, I^2^: 0.0%), vWF concentrations in trials with more than 60 patients decreased significantly (− 0.34, 95% CI: -0.56, − 0.12, I^2^: 60.9%).

**Conclusions:**

Overall, the current meta-analysis demonstrated that vitamin D supplementation to patients with metabolic syndrome and related disorders resulted in an improvement in vWF, but did not affect ICAM-1, VCAM-1, E-selectin and endothelin levels.

**Electronic supplementary material:**

The online version of this article (10.1186/s12986-018-0320-9) contains supplementary material, which is available to authorized users.

## Introduction

Atherosclerosis starts as endothelial activation, and endothelial dysfunction has been proposed to be the ultimate common pathway by which several risk factors result in vascular complications [1]. Vitamin D is a fat-soluble hormone that has endocrine, paracrine and autocrine functions [2]. Vitamin D deficiency and/or insufficiency may increase the risk of metabolic syndrome, type 2 diabetes mellitus (T2DM), hypertension, kidney disease and cardiovascular disease (CVD) through activating a pro-inflammatory cascade, which in turn may result in a rise in arterial stiffness and endothelial dysfunction [3, 4].

Previous studies have demonstrated that vitamin D administration improves endothelium-dependent vasodilation, a predictor of cardiovascular issues [5], among people with diabetes [6] and as well as healthy individuals with vitamin D deficiency [7]. In a meta-analysis study, vitamin D administration significantly improved flow-mediated dilation (FMD) [8]. Unlike, in another meta-analysis study conducted by Hussin et al. [9], vitamin D supplementation did not affect endothelial function. In addition, vitamin D supplementation to patients with T2DM did not affect vascular biomarkers including E-selectin and vWF [10]. VWF is a blood glycoprotein that is required for normal hemostasis [11]. It is deficient or defective in von Willebrand disease and is involved in a large number of other diseases, including thrombotic thrombocytopenic purpura, Heyde’s syndrome, and possibly hemolytic-uremic syndrome [11]. Increased circulating levels in a large number of cardiovascular, neoplastic, and connective tissue diseases are presumed to arise from adverse changes to the endothelium, and may contribute to an increased risk of thrombosis [12, 13]. However, there are no guiding values of markers related to endothelial function for clinical settings; control of these factors may reduce the progression of CVD events. The main mechanisms of function of vitamin D through which it may influence the atherosclerotic process have not been completely elucidated [14]. This may in part be through increased production of nitric oxide (NO), decreased oxidative damage, reduced gene expression of interleukin 6 (IL-6), and decreased circulating levels of vascular cell adhesion molecules (VCAM) and intracellular adhesion molecule (ICAM) [15]. Therefore, the results of those clinical trials are inclusive, with studies that favored vitamin D effects, oppositely to the others that did not confirm expected beneficial actions of this micronutrient. Discrepancies in findings might be the result of differences in study design, characteristics of study populations, dosage of vitamin D used and duration of the studies.

Despite several randomized controlled trials (RCTs), we are aware of no systematic review and meta-analysis of RCTs on the effect of vitamin D supplementation on endothelial activation among patients with metabolic syndrome and related disorders. This current meta-analysis was conducted to summarize the available evidence of RCTs to establish the effect of vitamin D supplementation on endothelial activation among patients with metabolic syndrome and related disorders.

## Methods

### Search strategy and selection studies

Related studies were systematically searched using several electronic databases: PubMed, Embase, the Cochrane Library, and Web of Science databases before 30th April 2018. We did not publish the review protocol. We conducted searches on gray literature using databases including institute for scientific and technical information (INIST) and the healthcare management information consortium (HMIC), also to find other unpublished studies, we contacted with experts and centers of related field. In addition, we searched the references lists of related trials to detect additional potential publications. Clinical trials retrieved that have examined the effect of vitamin D supplementation on endothelial activity based on the following MeSH and search terms: patients (“obese” OR “overweight” OR “T2DM” OR “acute coronary syndrome (ACS)” OR “myocardial infarction (MI)” OR “chronic kidney disease (CKD)” OR “metabolic syndrome (MetS)” OR “haemodialysis (HD)”), intervention (“ergocalciferol” OR “cholecalciferol” OR “vitamin D” OR “vitamin D_2_” OR “vitamin D_3_” OR “25-hydroxyvitamin D (25(OH)D)” AND “intake” OR “supplementation”), and outcomes [“von willebrand factor (vWF)” OR “VCAM-1” OR “ICAM-1” OR “E-selectin” OR “endothelin”]. Current systematic review was limited to clinical trials published in the English language.

### Data extraction and quality assessment

The following inclusion and exclusion criteria were applied to select clinical trials to be included in our meta-analysis: 1) human randomized clinical trials (either with parallel or crossover designs), 2) clinical trials that the administration of vitamin D supplements, and measured the mean changes of endothelial markers along with standard deviation (SD) at baseline and at the end of intervention in both treatment and control groups. The investigators were excluded articles that were case reports, the abstracts of congress without full text; and clinical trials that did not obtain minimum required score of quality assessment. Two independent authors (RT and MA) screened the titles and abstracts of the clinical trials to examine eligibility based on current criteria for inclusion. Then, we retrieved the full text of related clinical trials to evaluate with more details. In case of discrepancy, resolved by discussion with third author (ZA or STH).

### Data extraction and quality assessment

Two authors (RT and MA) independently extracted data and assessed the methodological quality of included clinical trials according to standard forms of excel software and the Cochrane Collaboration risk of bias tool, respectively. The following data were extracted from the selected RCTs: the first authors’ name, year of publication, geographic location of the study, total sample size of participants in intervention and control groups, study method, type of intervention, type of placebo, dosage of intervention, duration of follow-up, and the mean (SD) changes of on endothelial activation (including vWF, VCAM-1, ICAM-1, E-selectin, endothelin) at baseline and the end of intervention in both treatment and control groups. The Cochrane Collaboration risk of bias tool uses the following criteria to assess the methodological quality of clinical trials [16]: “random sequence generation, allocation concealment, blinding process and outcome assessment, and withdrawal of patients”. Discrepancies were resolved by discussion with third author (ZA or STH).

### Data analysis

Statistical analyses were conducted using Review Manager V.5.3 software (Cochrane Collaboration, Oxford, UK) and STATA version 12.0 (Stata Corp., College Station, TX). Heterogeneity between included primary studies was assessed using Cochrane Q test and I-square (I^2^) statistic. I^2^ > 50% with *P* <  0.05 indicates existing significant heterogeneity. We applied the random effects model to pool the information; otherwise, the fixed-effect model was conducted. Cohen statistics and DerSimonian and Laird method were used for estimation of standardized mean difference (SMD) with 95% confidence interval. Subgroup analyses were used to assess the potential variables that may have impacted the effects of vitamin D supplementation on endothelial activation. These potential variables contained: type of intervention (CKD vs. other vs. cardiac disease), dosage of vitamin D (≤400,000 vs. > 400,000 (IU)), duration of study (≤ 8 vs. > 8 weeks), sample size (≤60 vs. > 60 participants), and type of vitamin D (vitamin D3 vs. vitamin D2), BMI at baseline study (≥26 vs. ≤25 kg/m^2^) and baseline levels of 25(OH)D (≥15 vs. < 15 ng/mL). In addition, sensitivity analyses were used to assess the effect of each included primary study with leave-one-out method on reliability of the pooled SMDs. We examined the presence of publication bias using the formal Begg- and Egger’s statistics. *P* <  0.05 was considered as statistically significant.

## Results

**Fig. 1:**
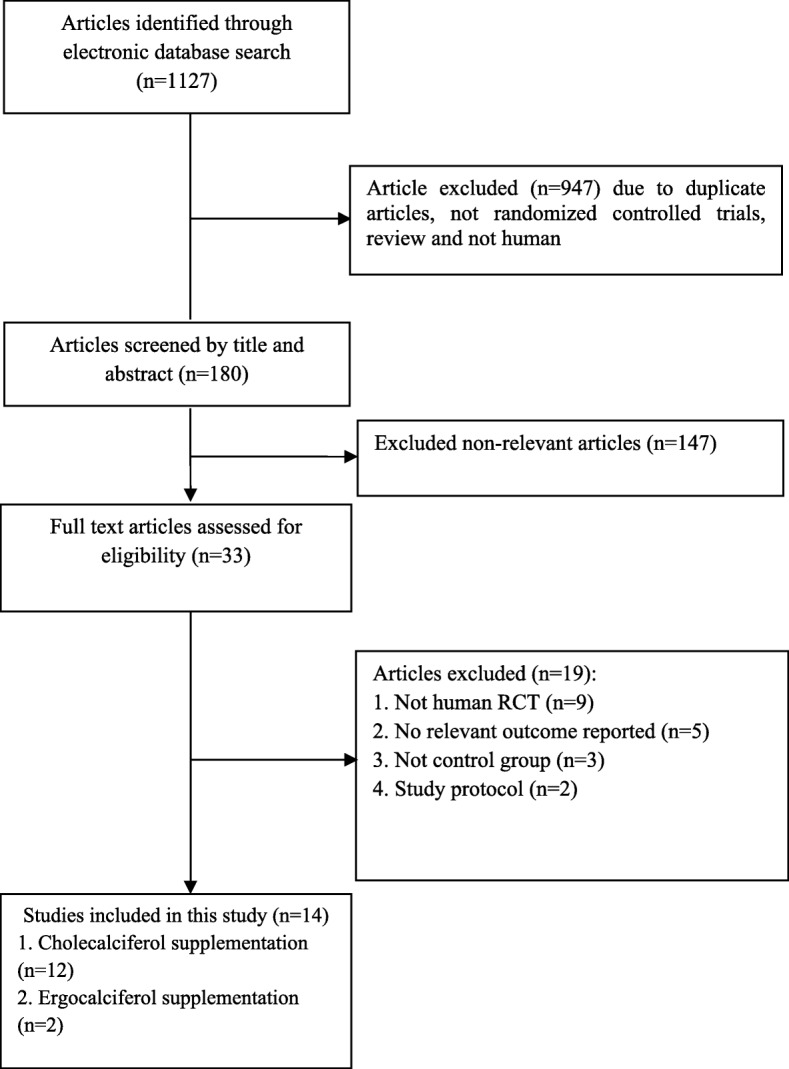
Literature search and review flowchart for selection of studies

Administered vitamin D in clinical trials included in our systematic review and meta-analysis was as powder or extract. An overall of 1127 studies were identified though our initial literatures using electronic search. After screening and assessing clinical trials with more detail, 14 trials were observed to be eligible for in the current study. Figure 1 shows the flow chart of the step by step study identification and selection process.

**Fig. 2:**
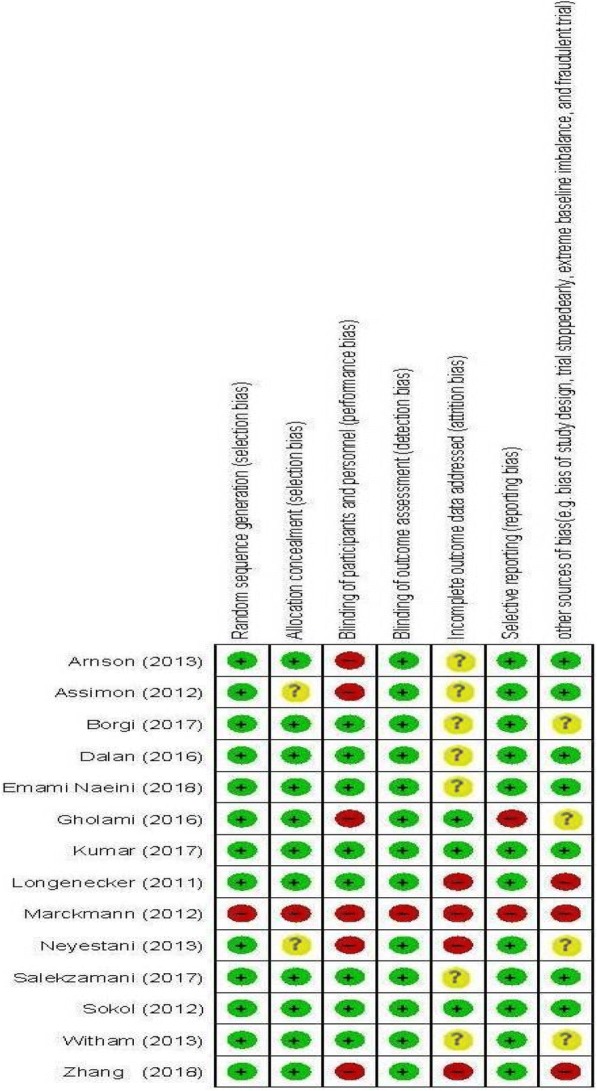
The methodological quality of included studies (risk of bias)

Characteristics of included clinical trials to our meta-analysis are summarized in Table 1. Included clinical trials were published between 2011 and 2018. This meta-analysis was applied on 1253 participants (with range 24–117) with various total sample size from 632 individuals in the intervention to 661 in the control group. Seven clinical trials have reported changes on vWF, 5 on ICAM-1, 7 on VCAM-1, 10 on E-selectin, 4 on endothelin. Dosage of vitamin D varied from 20,000 to about 1,300,000 IU. Duration of intervention among included clinical trials was between 5 days and 39 weeks. The results of quality assessment on included trials into our meta-analysis by using the Cochrane Collaboration tool based on authors’ judgments about each risk of bias item is presented in Fig. 2.

**Table 1:** The characteristics of included RCTs

Authors (Ref)	Publication year	Country	Intervention/control (sample size)	Duration (week)	Method of administration/Dosage	Patients	Weight change/BMI at baseline	Intervention/control (age participants)
Arnson at al. [19]	2013	Israel	25/25	5 days	Oral/4000 IU 25(OH)-vitamin D/day	Patients with acute coronary syndrome	NR/NR	61.6 ± 12.7, 59.7 ± 13.4
Witham (a) et al. [17]	2013	Scotland	36/39	6 months	Oral/ 100,000 IU vitamin D3 (0, 2 and 4 months)	Patients with a history of myocardial infarction	NR/NR	67.5 ± 10.6, 64.3 ± 9.8
Witham (b) et al. [17]	2013	Scotland	36/39	2 months	Oral/ 100,000 IU vitamin D3 (0 and 2 month)	Patients with a history of myocardial infarction	NR/NR	67.5 ± 10.6, 64.3 ± 9.8
Salekzamani et al. [35]	2017	Iran	36/35	16 weeks	Oral/ 50,000 IU/week for 16 weeks	Patients with metabolic syndrome	NR/33.37 ± 4.59	40.92 ± 5.95, 40.06 ± 4.83
Dalan et al. [10]	2016	Singapore	30/31	16 weeks	Oral/ Baseline 25(OH) D ≤ 20 ng/mL: four tablets of (4000 IU/day) Baseline 25(OH)D 21–30 ng/mL: 2000 IU/day	Patients with type 2 diabetes mellitus	NR/28.1 ± 5.9	54.8 ± 10.8, 52.2 ± 8.2
Neyestani (a) et al. [36]	2013	Iran	50/35	12 weeks	Oral/ Two 500 IU vitamin D for 12 weeks	Patients with type 2 diabetes with alleles Ff	No-significant/30.0 ± 5.0	52.4 ± 8.4, 52.6 ± 6.3
Neyestani (b) et al. [36]	2013	Iran	50/47	12 weeks	Oral/ Two 500 IU vitamin D for 12 weeks	Patients with type 2 diabetes with alleles Ff	No-significant/29.0 ± 4.2	52.4 ± 8.4, 52.6 ± 6.3
Neyestani © et al. [36]	2013	Iran	50/58	12 weeks	Oral/ Two 500 IU vitamin D for 12 weeks	Patients with type 2 diabetes with alleles Ff	No-significant/27.8 ± 4.7	52.4 ± 8.4, 52.6 ± 6.3
Sokol et al. [18]	2012	USA	45/45	12 weeks	Oral/ 50,000 IU ergocalciferol per week	Patients with coronary artery disease	NR/30.25 ± 6.9	56.96 ± 11.6, 55 ± 9.6
Longenecker et al. [20]	2011	USA	15/29	12 weeks	Oral/ Vitamin D3 4000 IU daily for 12 weeks	Patients with HIV-infected overweight	NR/27.5 ± 5.55	40 ± 10, 47 ± 8
Marckmann (a) et al. [37]	2012	Denmark	11/13	8 weeks	Oral/ 40,000 IU vitamin D3 every week for 8 weeks	Patients with chronic kidney disease	NR/25.25 ± 4.77	68 ± 21.68, 71 ± 20.40
Marckmann (b) et al. [37]	2012	Denmark	13/12	8 weeks	Oral/ 40,000 IU vitamin D3 every week for 8 weeks	Patients with chronic kidney disease	NR/25.25 ± 4.78	68 ± 21.68, 71 ± 20.40
Marckmann © et al. [37]	2012	Denmark	26/26	8 weeks	Oral/ 40,000 IU vitamin D3 every week for 8 weeks	Patients with chronic kidney disease	NR/25.25 ± 4.79	68 ± 21.68, 71 ± 20.40
Kumar et al. [38]	2017	India	59/58	16 weeks	Oral/ Cholecalciferol (300,000 IU) for 8 weeks	Patients with nondiabetic chronic kidney disease stage 3–4	NR/23.51 ± 2.79	45.20 ± 11.61, 43.17 ± 11.79
Emami Naeini et al. [39]	2018	Iran	32/32	16 weeks	Oral/ 50,000 IU vitamin D per week for 12 weeks	End-stage renal disease patients	NR/26.8 ± 7	62 ± 21, 60 ± 19
Assimon et al. [40]	2012	USA	20/20	39 weeks	Oral/ 33,125 ± 17,805 IU/week for an average duration of 39.2 ± 28.3 weeks	Haemodialysis patients	NR/29.9 ± 6.5	64.4 ± 15.6, 68.1 ± 15.9
Zhang et al. [21]	2018	China	46/25	12 weeks	Oral/ 50,000 IU cholecalciferol once a week for 12 weeks	Patients with non-dialysis chronic kidney disease	NR/22.7 ± 2.2	61.8 ± 16.6, 55.5 ± 14.7
Gholami et al. [41]	2016	Iran	40/20	4 weeks	Intramuscular/ Single dose of 300,000 IU vitamin D3	Patients with venous thromboembolism	NR/NR	53.2 ± 17.3
Borgi et al. [22]	2017	USA	41/43	8 weeks	Oral/ 50,000 IU/week ergocalciferol for 4 weeks	Overweight or obese adults	NR/33.9 ± 5.8	35 ± 11, 39 ± 13

NR, not reported

### Pooled effects of vitamin D supplementation on endothelial activation

**Fig. 3:**
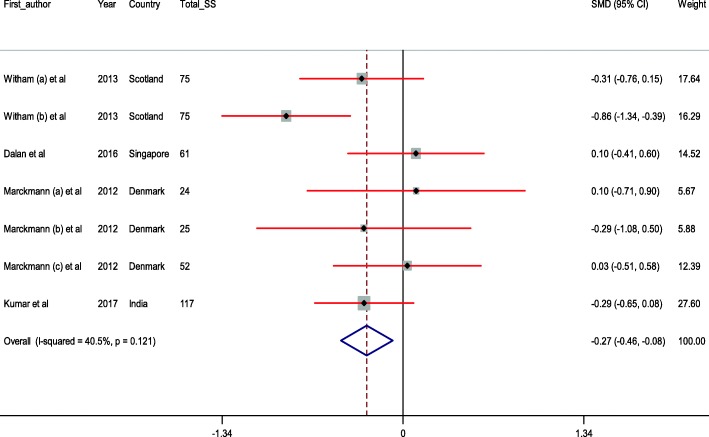
Meta-analysis endothelial activation standardized mean differences estimates for vWF in vitamin D and placebo groups (CI = 95%)

The forest plots for the effect of vitamin D supplementation on endothelial activation are shown in Additional file 1 and Fig. 3.

The findings showed that vitamin D administration significantly decreased vWF (SMD -0.27; 95% CI, − 0.46, − 0.08; *P* = 0.006; I^2^:40.5%) (Fig. 3). However, we found no significant impact of vitamin D supplementation on ICAM-1 (SMD -1.96; 95% CI, − 4.02, 0.09; *P* = 0.06; I^2^:97.4%), VCAM-1 (SMD -0.50; 95% CI, − 1.19, 0.19; *P* = 0.15; I^2^:91.2%), E-selectin (SMD -0.04; 95% CI, − 0.36, 0.28; *P* = 0.81; I^2^:78.8%), and endothelin levels (SMD -0.49; 95% CI, − 1.18, 0.19; P = 0.15; I^2^:90.5%) (Additional file 1).

Table 2 summarizes all meta-analyses findings of the study participant included RCTs with data before and after the intervention and placebo groups.

**Table 2:** The effects of vitamin D supplementation on endothelial activation

Variable	Number of study	Standardized mean difference	CI 95%	Heterogeneity		
				I^2^ (%)	Q	*P*-value
VWF						
Change intervention group vs. placebo group	7	-0.27	−0.46, − 0.08	40.5	10.08	0.12
ICAM-1						
Intervention group (after vs. before)	4	−0.19	− 0.45, 0.06	18.9	3.70	0.29
Placebo group (after vs. before)	4	3.51	1.28, 5.73	97.6	124.47	< 0.001
Change intervention group vs. placebo group	5	−1.96	−4.02, 0.09	97.4	153.96	< 0.001
VCAM-1						
Intervention group (after vs. before)	6	−0.24	−0.44, −0.03	0.0	1.18	0.94
Placebo group (after vs. before)	6	−0.08	− 0.49, 0.33	76.5	21.32	0.001
Change intervention group vs. placebo group	7	−0.50	−1.19, 0.19	91.2	68.54	< 0.001
E-selectin						
Intervention group (after vs. before)	8	−0.25	−0.41, − 0.08	0.0	6.15	0.52
Placebo group (after vs. before)	8	−0.10	−0.37, 0.17	66.0	20.56	0.004
Change intervention group vs. placebo group	10	−0.04	−0.36, 0.28	78.8	42.49	< 0.001
Endothelin						
Intervention group (after vs. before)	4	−0.45	− 0.66, − 0.24	40.1	5.01	0.17
Placebo group (after vs. before)	4	0.11	−0.10, 0.31	0.0	2.98	0.39
Change intervention group vs. placebo group	4	−0.49	−1.18, 0.19	90.5	31.46	< 0.001

*VWF* von willebrand factor, *ICAM-1* intercellular adhesion molecule 1, *VCAM-1* vascular cell adhesion molecule 1

### Subgroup and sensitivity analyses

Because of presence heterogeneity, the authors used subgroup analyses to assessed source of heterogeneity based on suspected variables. The results of subgroup analyses based on dosage of vitamin D (≤4000 vs. > 4000 IU/day), type of vitamin D (vitamin D3 vs. vitamin D2), BMI at baseline (≥26 vs. ≤25 kg/m^2^), and baseline levels of 25(OH) D (≥15 vs. < 15 ng/mL) are presented in Additional file 2. Overall, there were no significant changes in associations between dosages, type of vitamin D used, and BMI of participants and endothelial activations. We were not able to conduct a subgroup analyses based on method of vitamin D administration (oral or intramuscular), as there was only one study used injection method. However, our sensitivity analyses showed no effect when we exclude this study. Compared with the trials on participants with CKD diseases (− 0.17, 95% CI: -0.43, 0.10, I^2^: 0.0%) or other diseases (0.10, 95% CI: -0.41, 0.60, I^2^: 0.0%), vitamin D consumption in clinical trials with cardiac diseases participants (− 0.57, 95% CI: -0.90, − 0.25, I^2^: 63.8%) significantly decreased vWF concentrations. The pooled data from trials of vitamin D supplementation with dosage of ≤4000 IU/day (− 0.37, 95% CI: -0.65, − 0.10, I^2^: 73.5%) significantly reduced vWF concentrations, while there was no effect of vitamin D supplementation on vWF concentrations among trials with the dosage of intervention > 4000 IU/day (− 0.17, 95% CI: -0.43, 0.10, I^2^: 0.0%). VWF concentrations significantly reduced in pooled data from trials with duration study ≤8 weeks (− 0.37, 95% CI: -0.67, − 0.07, I^2^: 60.6%), but there was no effect of vitamin D supplementation on vWF concentrations among trials with > 8 weeks (− 0.20, 95% CI: -0.45, 0.05, I^2^: 0.0%). While there was no effect of vitamin D supplementation on vWF concentrations among trials with total sample size of ≤60 patients (− 0.03, 95% CI: -0.42, 0.36, I^2^: 0.0%), vWF concentrations in trials with more than 60 patients decreased significantly (− 0.34, 95% CI: -0.56, − 0.12, I^2^: 60.9%). The pooled data from trials with baseline levels of 25(OH)D ≥ 15 ng/mL (− 0.34, 95% CI: -0.56, − 0.12, I^2^: 60.9%) significantly decreased vWF, but there was no effect of vitamin D supplementation on vWF concentrations among trials with < 15 ng/mL (− 0.03, 95% CI: -0.42, 0.36, I^2^: 0.0%).

ICAM-1 levels significantly decreased following vitamin D administration in pooled data from clinical trials with dosage > 4000 IU (− 10.06, 95% CI: -15.94, − 4.18, I^2^: 98.6%), while there was no effect of vitamin D supplementation on ICAM-1 concentrations among trials with the dosage ≤4000 IU/day (− 0.09, 95% CI: -0.61, 0.42, I^2^: 55.4%) **(**Additional file 2). In addition, compared with duration study ≤8 weeks (− 0.39, 95% CI: -0.95, 0.17, I^2^: 0.0%), ICAM-1 levels significantly decreased following vitamin D administration in pooled data from clinical trials with duration > 8 weeks (− 3.43, 95% CI: -6.43, − 0.42, I^2^: 98.1%). Also, compared with trials with vitamin D2 intervention (− 1.14, 95% CI: -3.70, 1.42, I^2^: 96.7%), ICAM-1 levels in trials with vitamin D3 intervention significantly decreased (− 5.93, 95% CI: -10.43, − 1.42, I^2^: 98.4%).

E-selectin concentrations significantly decreased in pooled data from clinical trials with dosage of vitamin D ≤ 400,000 IU (− 0.15, 95% CI: -0.35, 0.05, I^2^: 25.3%) compared with > 400,000 IU (0.25, 95% CI: -0.93, 1.42, I^2^: 93.7%) (Additional file 2). E-selectin concentrations significantly decreased in pooled data from clinical trials with baseline levels of 25(OH)D < 15 (− 0.35, 95% CI: -0.57, − 0.13, I^2^: 40.79%) compared with ≥15 ng/mL (0.17, 95% CI: -0.32, 0.67, I^2^: 83.9%).

For VCAM-1 concentrations, clinical trials were found no significant changes between the intervention and control groups by suspected variables using subgroup analyses (Additional file 2).

In pooled data from clinical trials with duration > 8 weeks (− 0.84, 95% CI: -1.08, − 0.60, I^2^: 0.0%), endothelin concentrations significantly decreased, but endothelin concentrations significantly increased in clinical trials with duration ≤8 weeks (0.58, 95% CI: 0.14, 1.01, I^2^: 0.0%) (Additional file 2). Also, endothelin concentrations significantly decreased in pooled data from clinical trials with total sample size > 60 participants (− 0.84, 95% CI: -1.08, − 0.60, I^2^: 0.0), while endothelin concentrations significantly increased in clinical trials with total sample size ≤60 patients (0.58, 95% CI: 0.14, 1.01, I^2^: 0.0). Similarly, endothelin levels significantly increased (0.58, 95% CI: 0.14, 1.01, I^2^: 0.0%) in trials using vitamin D2 supplements, while endothelin levels decreased significantly (− 0.84, 95% CI: -1.08, − 0.60, I^2^: 0.0%) in trials administrated vitamin D3. The pooled data from trials with dosage ≤4000 IU/day (− 0.91, 95% CI: -1.22, − 0.61, I^2^: 0.0%) significantly reduced endothelin concentrations, but there was no effect of vitamin D supplementation on endothelin concentrations among trials with the dosage > 4000 IU/day (− 0.07, 95% CI: -1.34, 1.19, I^2^: 94.6%).

In sensitivity analysis, to evaluate the effect of each of study on the strength of association between vitamin D supplementation and endothelial activation, the pooled SMD was presented pre and post excluding one by one clinical trial from our meta-analysis. According to sensitivity analyses findings, we found that after omitting the data from Witham (b) et al. [17] for vWF (SMD -0.15; 95% CI, − 0.36, 0.05), after omitting the data from Sokol et al. [18] (SMD -3.88; 95% CI, − 7.06, − 0.70), Arnson et al. [19] (SMD -3.42; 95% CI, − 6.40, − 0.42), and Longenecker et al. [20] (SMD -3.29; 95% CI, − 6.16, − 0.43) for ICAM-1, after omitting the data from Zhang et al. [21] (SMD -0.75; 95% CI, − 1.34, − 0.16) for VCAM-1, after omitting the data from Zhang et al. [21] (SMD -0.20; 95% CI, − 0.36, − 0.03) for E-selectin, after omitting the data from Borgi et al. [22] (SMD -0.83; 95% CI, − 1.07, − 0.59) for Endothelin, there were significant effect between pre- and post-sensitivity pooled SMD for their endothelial markers (Additional file 3).

### Publication Bias

According to the findings of Egger and Begg statistics, there was no evidence of publication bias across trials for vWF (P Egger’s test = 0.30, P Begg’s test = 0.45), VCAM-1 (P_Eg_ = 0.23, P_Be_ = 0.17), and E-selectin (P_Eg_ = 0.25, P_Be_ = 0.09), and Endothelin (P_Eg_ = 0.78, P_Be_ = 1.0). Because there was evidence of publication bias on ICAM-1 (P_Eg_ = 0.43, P_Be_ = 0.17), the authors performed non parametric method (Duval and Tweedie) to assess the findings of censored trials. Results indicated that summary effect size on ICAM-1 not significantly changed between before and after including censored trials.

## Discussion

This systematic review and meta-analysis is the first report of the effect of vitamin D supplementation on endothelial activation among patients with metabolic syndrome and related disorders. This meta-analysis showed that taking vitamin D significantly improved vWF, but did not affect other markers related to endothelial activation among patients with metabolic syndrome and related disorders. In the current study, patients with metabolic syndrome and related disorders were included that are varied. Endothelial activation is one of the main pathophysiological causes and is part of different chronic conditions including obesity, T2DM, CVD, stroke, fatty liver, and other metabolic diseases. Therefore, due to similar metabolic status, we included a board range of chronic diseases in the current meta-analysis. However, the effectiveness of vitamin D supplementation may vary with the type of disease. For example, CKD is a very vitamin D sensitive state [18]. To address this limitation, we have conducted subgroup analysis based on type of metabolic diseases to decrease heterogeneity (e.g. CKD vs. other vs. cardiac disease). Extensive sensitivity analyses were also conducted; for example, excluding those that had different outcomes, none of these omissions significantly altered overall estimates, which suggests that our estimates are reliable.

Cardiovascular factors are the leading cause of mortality in diseases related to metabolic disorders [23]. This meta-analysis demonstrated that vitamin D supplementation to patients with metabolic syndrome and related disorders resulted in a significant decrease in vWF, but did not affect other markers of endothelial activation. Observed effects for ICAM from clinical trials with duration > 8 weeks compared with ≤8 weeks were significant, while for vWF from clinical trials with duration ≤8 weeks compared with > 8 weeks were significant; that was against our hypothesis. This may have few reasons. The limited number of studies included in the subgroups analyses may be one possible explanation for such discrepancy. The larger RCTs are required to obtain more reliable conclusion. In addition, the subjects recruited in observational studies had different baseline levels of vWF. Thus, we assumed that early intervention with vitamin D in patients with metabolic syndrome and related disorders may be important; while the beneficiary effect of vitamin D on vWF may be increased when individuals have longer duration of supplementation and similar baseline levels of vWF. Also, we should acknowledge and distinguish between “statistical significance” which implies that the difference seen in the sample also exists in the population with “clinical significance” which implies that the difference between effectiveness is clinically important, and it is possible that clinical practice will change if such a difference is seen. Although statistical significance is used to inform clinical significance, but clinical significance cannot necessarily be inferred from statistical significance, and statistical significance cannot be inferred from clinical significance. Hypovitaminosis D is a common problem affecting over 40% of the United States population [24]. Vitamin D deficiency has been independently correlated with elevated risk of CVD, severity of coronary atherosclerosis, and all-cause mortality [25, 26]. Few studies have reported high levels of vWF in patients related to metabolic disorders. Lu et al. [27] demonstrated increased levels of vWF in people with chronic kidney disease (CKD) compared with healthy subjects. In another study, diabetic patients with chronic hemodialysis had elevated vWF levels [28]. Several RCTs have been performed in the last decade to assess the impacts of vitamin D administration on endothelial function among patients with metabolic syndrome and related disorders. In a study, using a single dose of 100,000 vitamin D to patients with T2DM, there was an improvement in endothelial function by 2.5% at 8 weeks [6]. However, few meta-analyses studied have evaluated the effects of vitamin D supplementation on endothelial function, data on the effects of vitamin D supplementation on endothelial activity are scarce. In a meta-analysis study, vitamin D intake significantly improved FMD [8]. In addition, a significant improvement of endothelial vasomotor and secretory functions was seen following the supplementation of vitamin D in people with CKD [29]. Unlike, in another meta-analysis study conducted by Hussin et al. [9], vitamin D administration did not influence endothelial function. Moreover, vitamin D supplementation for 6 months to people with a history of myocardial infarction did not improve markers of vascular function [17]. In another meta-analysis study, vitamin D supplementation did not improve FMD; however, vitamin D improved FMD in studies that lasted < 16 weeks [30].

The different dosages of vitamin D used, means of supplementation and type of vitamin D used are some of the possible reasons that may be correlated with the different results in VWF among these previous studies. Although the direct route of the endothelial activation is unknown; several mechanisms have been proposed by which vitamin D supplements could improve endothelial activation. Vitamin D receptors have been recognized in several cell types, such as vascular smooth muscle cells, endothelial cells and cardiac myocytes [31]. Vitamin D intake may possibly reduce proliferation of vascular smooth muscle, dysregulate systemic vascular calcium metabolism; reduce vascular resistance, downregulate pro-inflammatory markers, upregulate anti-inflammatory markers and decreases blood pressure through regulation of the rennin-angiotensin system [32, 33]. Synthesis of 1, 25-dihydroxyvitamin D3 by human endothelial cells may play at the local levels to regulate the effects of inflammatory markers on the vasculature [34]. However, findings of epidemiological studies suggest that vitamin D administration has a positive effect on FMD and may reduce CVD risk and related complications.

The current study had a few limitations. Various doses of vitamin D used were administered for intervention in the included studies. We were unable to evaluate the dose-response association between supplementation and endothelial markers. One of the major limitations of the study was the inclusion of studies with relatively small sample size that could influence type-2 statistical error. In addition, in vitamin D supplementation trials, considering the baseline 25(OH)D level is important. High levels of 25(OH)D before supplementation may attenuate the supplementation effect. Some studies did not report baseline levels of 25(OH)D, therefore we could not include in subgroup analysis.

## Conclusions

Overall, the current meta-analysis demonstrated that vitamin D supplementation to patients with metabolic syndrome and related disorders resulted in an improvement in vWF, but did not affect ICAM-1, VCAM-1, E-selectin and endothelin levels

## Additional files

Additional file 1: Meta-analysis endothelial activation standardized mean differences estimates for (A) for ICAM-1, (B) for VCAM-1, (C) for E-selectin and (D) for endothelin in vitamin D and placebo groups (CI = 95%). (PPTX 60 kb)

Additional file 2: The association between vitamin D supplementation on endothelial activation based on subgroup analysis. (DOC 128 kb)

Additional file 3: The association between vitamin D supplementation and endothelial activation based on sensitivity analysis. (DOCX 13 kb)

## Abbreviations

ICAM-1: Intercellular adhesion molecule 1
VCAM-1: Vascular cell adhesion molecule 1
VWF: Von willebrand factor

## References

[1] Cohn JN, Quyyumi AA, Hollenberg NK, Jamerson KA (2004). Surrogate markers for cardiovascular disease: functional markers. Circulation.

[2] Vanchinathan V, Lim HW (2012). A dermatologist’s perspective on vitamin D. Mayo Clin Proc.

[3] Geleijnse JM (2011). Vitamin D and the prevention of hypertension and cardiovascular diseases: a review of the current evidence. Am J Hypertens.

[4] Vaidya A, Forman JP (2012). Vitamin D and vascular disease: the current and future status of vitamin D therapy in hypertension and kidney disease. Curr Hypertens Rep.

[5] Mazidi M, Michos ED, Banach M (2017). The association of telomere length and serum 25-hydroxyvitamin D levels in US adults: the National Health and nutrition examination survey. Arch Med Sci.

[6] Sugden JA, Davies JI, Witham MD, Morris AD, Struthers AD (2008). Vitamin D improves endothelial function in patients with type 2 diabetes mellitus and low vitamin D levels. Diabet Med.

[7] Tarcin O, Yavuz DG, Ozben B, Telli A, Ogunc AV, Yuksel M (2009). Effect of vitamin D deficiency and replacement on endothelial function in asymptomatic subjects. J Clin Endocrinol Metab.

[8] Mazidi M, Karimi E, Rezaie P, Vatanparast H (2017). The impact of vitamin D supplement intake on vascular endothelial function; a systematic review and meta-analysis of randomized controlled trials. Food Nutr Res.

[9] Hussin AM, Ashor AW, Schoenmakers I, Hill T, Mathers JC, Siervo M (2017). Effects of vitamin D supplementation on endothelial function: a systematic review and meta-analysis of randomised clinical trials. Eur J Nutr.

[10] Dalan R, Liew H, Assam PN, Chan ES, Siddiqui FJ, Tan AW (2016). A randomised controlled trial evaluating the impact of targeted vitamin D supplementation on endothelial function in type 2 diabetes mellitus: the DIMENSION trial. Diab Vasc Dis Res.

[11] Sadler JE (1998). Biochemistry and genetics of von Willebrand factor. Annu Rev Biochem.

[12] Ristic GG, Subota V, Lepic T, Stanisavljevic D, Glisic B, Ristic AD (2015). Subclinical atherosclerosis in patients with rheumatoid arthritis and low cardiovascular risk: the role of von Willebrand factor activity. PLoS One.

[13] Mannucci PM, Vanoli M, Forza I, Canciani MT, Scorza R (2003). Von Willebrand factor cleaving protease (ADAMTS-13) in 123 patients with connective tissue diseases (systemic lupus erythematosus and systemic sclerosis). Haematologica.

[14] Pearson TA, Mensah GA, Alexander RW, Anderson JL, Cannon RO, Criqui M (2003). Markers of inflammation and cardiovascular disease: application to clinical and public health practice: a statement for healthcare professionals from the Centers for Disease Control and Prevention and the American Heart Association. Circulation.

[15] Dalan R, Liew H, Tan WKA, Chew DE, Leow MK-S (2014). Vitamin D and the endothelium: basic, translational and clinical research updates. IJC Metabolic & Endocrine.

[16] Higgins JP, Altman DG, Gotzsche PC, Juni P, Moher D, Oxman AD (2011). The Cochrane Collaboration’s tool for assessing risk of bias in randomised trials. BMJ.

[17] Witham MD, Dove FJ, Khan F, Lang CC, Belch JJ, Struthers AD (2013). Effects of vitamin D supplementation on markers of vascular function after myocardial infarction—a randomised controlled trial. Int J Cardiol.

[18] Sokol SI, Srinivas V, Crandall JP, Kim M, Tellides G, Lebastchi AH (2012). The effects of vitamin D repletion on endothelial function and inflammation in patients with coronary artery disease. Vasc Med.

[19] Arnson Y, Itzhaky D, Mosseri M, Barak V, Tzur B, Agmon-Levin N (2013). Vitamin D inflammatory cytokines and coronary events: a comprehensive review. Clin Rev Allergy Immunol.

[20] Longenecker CT, Hileman CO, Carman TL, Ross AC, Seydafkan S, Brown TT (2012). Vitamin D supplementation and endothelial function in vitamin D deficient HIV-infected patients: a randomized placebo-controlled trial. Antivir Ther.

[21] Zhang Q, Zhang M, Wang H, Sun C, Feng Y, Zhu W (2018). Vitamin D supplementation improves endothelial dysfunction in patients with non-dialysis chronic kidney disease. Int Urol Nephrol.

[22] Borgi L, McMullan C, Wohlhueter A, Curhan GC, Fisher ND, Forman JP (2017). Effect of vitamin D on endothelial function: a randomized, double-blind, placebo-controlled trial. Am J Hypertens.

[23] Sharma A, de Souza BF, Sun JL, Thomas L, Haffner S, Holman RR (2017). Noncardiovascular deaths are more common than cardiovascular deaths in patients with cardiovascular disease or cardiovascular risk factors and impaired glucose tolerance: insights from the Nateglinide and valsartan in impaired glucose tolerance outcomes research (NAVIGATOR) trial. Am Heart J.

[24] Forrest KY, Stuhldreher WL (2011). Prevalence and correlates of vitamin D deficiency in US adults. Nutr Res.

[25] Wang L, Song Y, Manson JE, Pilz S, Marz W, Michaelsson K (2012). Circulating 25-hydroxy-vitamin D and risk of cardiovascular disease: a meta-analysis of prospective studies. Circ Cardiovasc Qual Outcomes.

[26] Dziedzic EA, Przychodzen S, Dabrowski M (2016). The effects of vitamin D on severity of coronary artery atherosclerosis and lipid profile of cardiac patients. Arch Med Sci.

[27] Taniguchi S, Hashiguchi T, Ono T, Takenouchi K, Nakayama K, Kawano T (2010). Association between reduced ADAMTS13 and diabetic nephropathy. Thromb Res.

[28] Cohen-Hagai K, Rashid G, Einbinder Y, Ohana M, Benchetrit S, Zitman-Gal T (2017). Effect of vitamin D status on von Willebrand factor and adamts13 in diabetic patients on chronic hemodialysis. Ann Lab Med.

[29] Chitalia N, Ismail T, Tooth L, Boa F, Hampson G, Goldsmith D (2014). Impact of vitamin D supplementation on arterial vasomotion, stiffness and endothelial biomarkers in chronic kidney disease patients. PLoS One.

[30] Stojanovic M, Radenkovic M (2015). Vitamin D versus placebo in improvement of endothelial dysfunction: a meta-analysis of randomized clinical trials. Cardiovasc Ther.

[31] Wang TJ, Pencina MJ, Booth SL, Jacques PF, Ingelsson E, Lanier K (2008). Vitamin D deficiency and risk of cardiovascular disease. Circulation.

[32] Zittermann A, Schleithoff SS, Koerfer R (2005). Putting cardiovascular disease and vitamin D insufficiency into perspective. Br J Nutr.

[33] Reddy Vanga S, Good M, Howard PA, Vacek JL (2010). Role of vitamin D in cardiovascular health. Am J Cardiol.

[34] Zehnder D, Bland R, Chana RS, Wheeler DC, Howie AJ, Williams MC (2002). Synthesis of 1,25-dihydroxyvitamin D(3) by human endothelial cells is regulated by inflammatory cytokines: a novel autocrine determinant of vascular cell adhesion. J Am Soc Nephrol.

[35] Salekzamani S, Bavil AS, Mehralizadeh H, Jafarabadi MA, Ghezel A, Gargari BP (2017). The effects of vitamin D supplementation on proatherogenic inflammatory markers and carotid intima media thickness in subjects with metabolic syndrome: a randomized double-blind placebo-controlled clinical trial. Endocrine.

[36] Neyestani TR, Djazayery A, Shab-Bidar S, Eshraghian MR, Kalayi A, Shariatzadeh N (2013). Vitamin D receptor Fok-I polymorphism modulates diabetic host response to vitamin D intake: need for a nutrigenetic approach. Diabetes Care.

[37] Marckmann P, Agerskov H, Thineshkumar S, Bladbjerg EM, Sidelmann JJ, Jespersen J (2012). Randomized controlled trial of cholecalciferol supplementation in chronic kidney disease patients with hypovitaminosis D. Nephrol Dial Transplant.

[38] Kumar V, Yadav AK, Lal A, Kumar V, Singhal M, Billot L (2017). A randomized trial of vitamin D supplementation on vascular function in CKD. J Am Soc Nephrol.

[39] Naeini AE, Moeinzadeh F, Vahdat S, Ahmadi A, Hedayati ZP, Shahzeidi S (2017). The effect of vitamin D administration on intracellular adhesion molecule-1 and vascular cell adhesion molecule-1 levels in hemodialysis patients: a placebo-controlled, double-blinded clinical trial. J Res Pharm Pract.

[40] Assimon MM, Salenger PV, El-Fawal HA, Mason DL (2012). Nutritional vitamin D supplementation in haemodialysis: a potential vascular benefit?. Nephrology (Carlton).

[41] Gholami K, Talasaz AH, Entezari-Maleki T, Salarifar M, Hadjibabaie M, Javadi MR (2016). The effect of high-dose vitamin D3 on soluble P-selectin and hs-CRP level in patients with venous thromboembolism: a randomized clinical trial. Clin Appl Thromb Hemost.

