# Supplementary material for: The effects of vitamin D supplementation on endothelial activation among patients with metabolic syndrome and related disorders: a systematic review and meta-analysis of randomized controlled trials
Source: Nutr Metab (Lond). 2018 Nov 29;15:85. doi: 10.1186/s12986-018-0320-9 (PMC6267828; doi:10.1186/s12986-018-0320-9)
Supplement: Supplementary file 1 — Meta-analysis endothelial activation standardized mean differences estimates for (A) for ICAM-1, (B) for VCAM-1, (C) for E-selectin and (D) for endothelin in vitamin D and placebo groups (CI = 95%). (PPTX 60 kb) [file 12986_2018_320_MOESM1_ESM.pptx]

## Slide 1
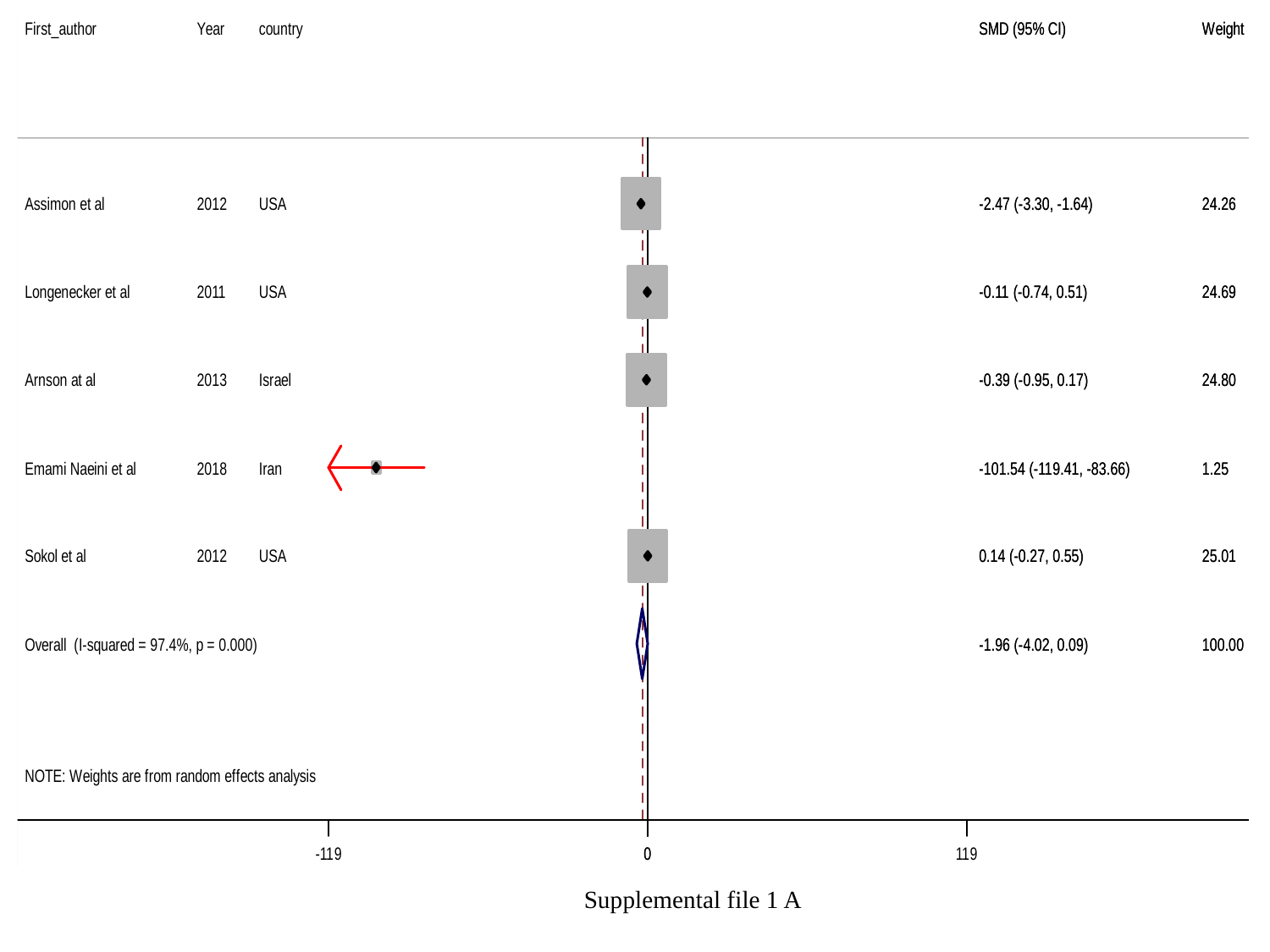

Supplemental file 1 A

## Slide 2
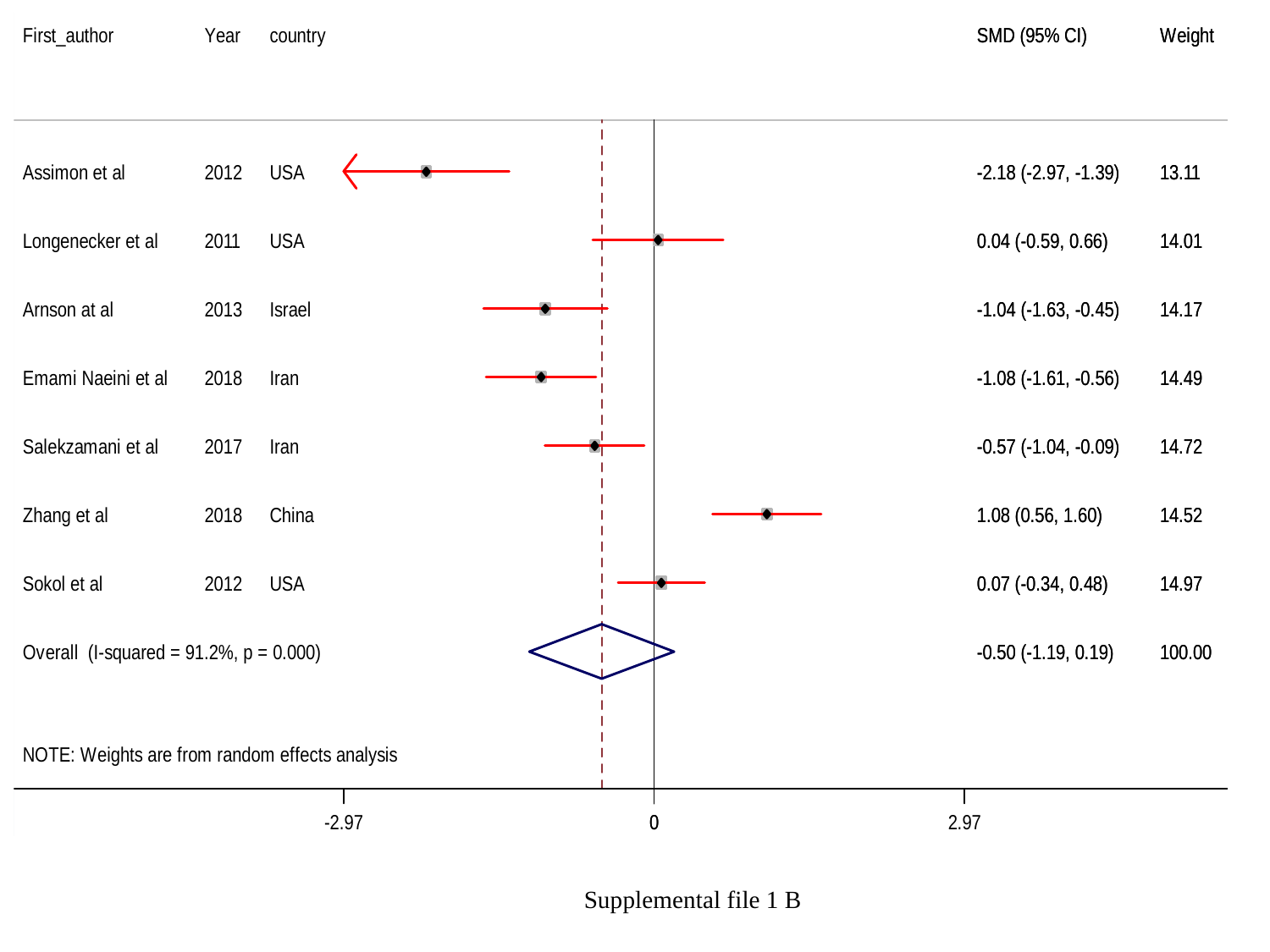

Supplemental file 1 B

## Slide 3
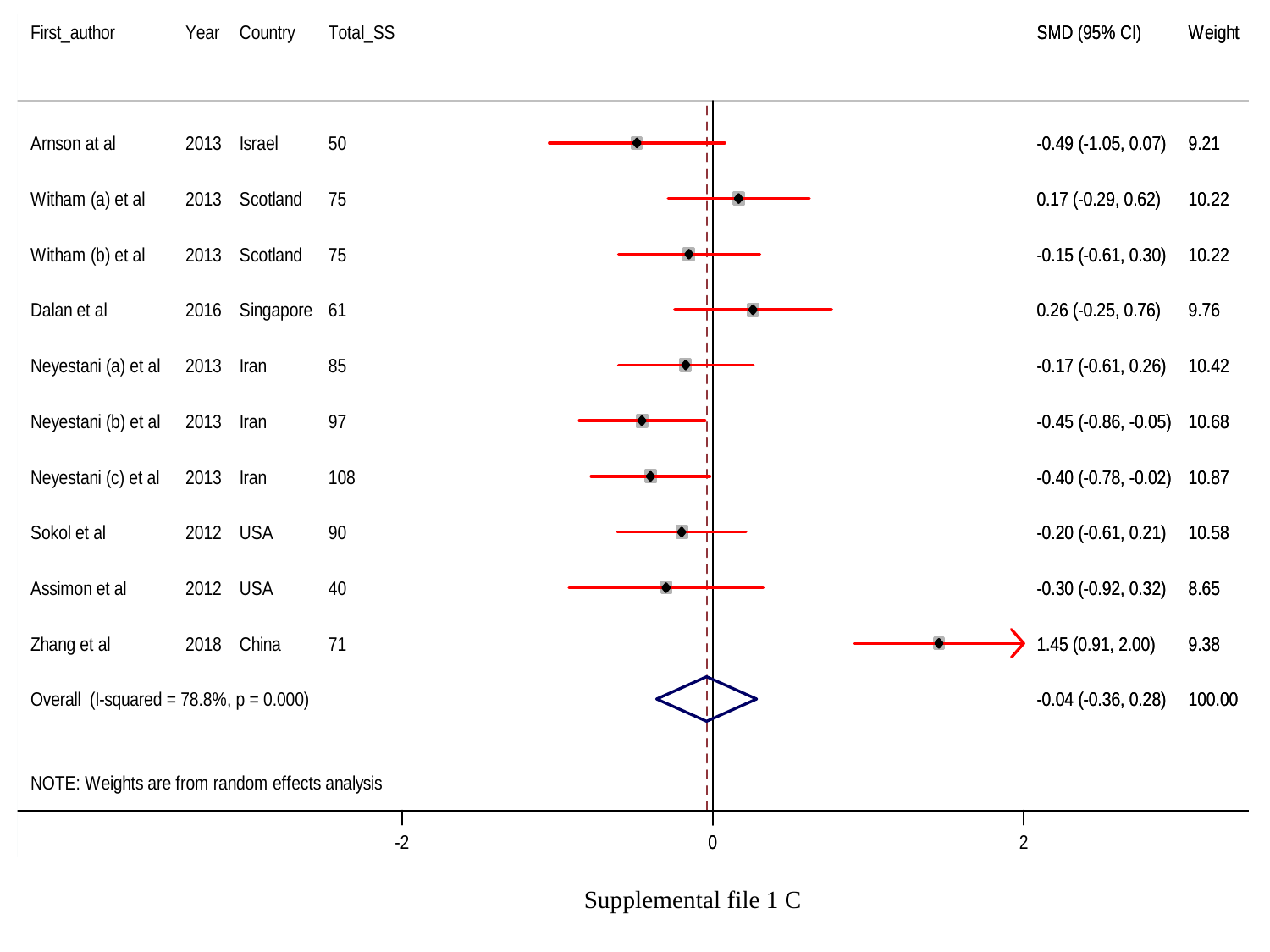

Supplemental file 1 C

## Slide 4
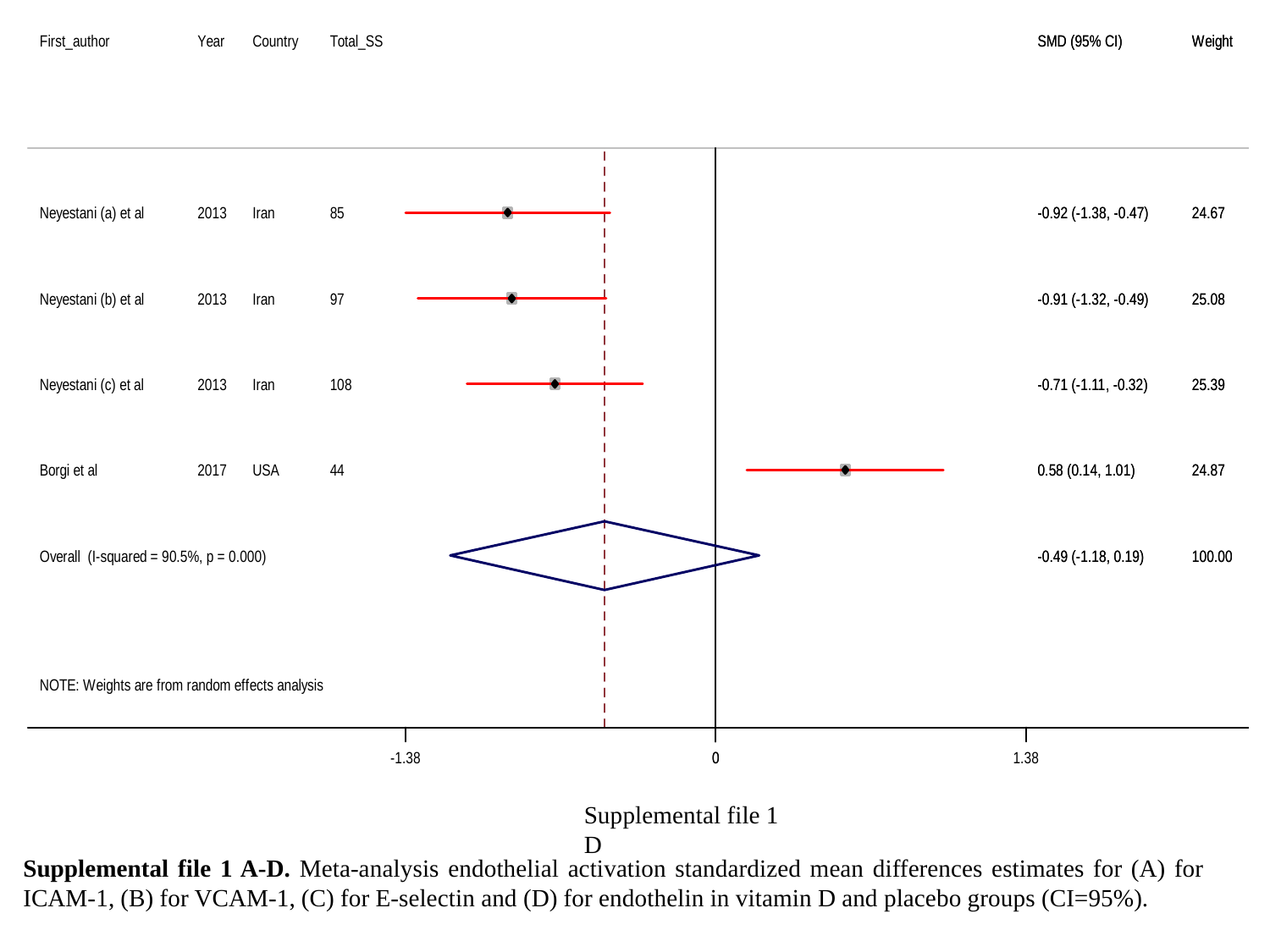

Supplemental file 1 D
Supplemental file 1 A-D. Meta-analysis endothelial activation standardized mean differences estimates for (A) for ICAM-1, (B) for VCAM-1, (C) for E-selectin and (D) for endothelin in vitamin D and placebo groups (CI=95%).
