# Supplementary material for: The effects of vitamin D supplementation on endothelial activation among patients with metabolic syndrome and related disorders: a systematic review and meta-analysis of randomized controlled trials
Source: Nutr Metab (Lond). 2018 Nov 29;15:85. doi: 10.1186/s12986-018-0320-9 (PMC6267828; doi:10.1186/s12986-018-0320-9)
Supplement: Supplementary file 2 — The association between vitamin D supplementation on endothelial activation based on subgroup analysis. (DOC 128 kb) [file 12986_2018_320_MOESM2_ESM.doc]

**Supplemental file 2**. The association between vitamin D supplementation on endothelial activation based on subgroup analysis

| Variables | | Number of SMD  included | Subgroups | SDM  (random effect) | 95% CI | I^2^ (%) | overall  I^2^(%) |
| --- | --- | --- | --- | --- | --- | --- | --- |
| VWF | Type of intervention | 4 | CKD | -0.17 | -0.43, 0.10 | 0.0 | 40.5 |
|  |  | 1 | Other | 0.10 | -0.41, 0.60 | - |  |
|  |  | 2 | Cardiac disease | -0.57 | -0.90, -0.25 | 63.8 |  |
|  | Dosage of vitamin D (IU/day) | 3 | ≤4,000 | -0.37 | -0.65, -0.10 | 73.5 |  |
|  |  | 4 | >4,000 | -0.17 | -0.43, 0.10 | 0.0 |  |
|  | Duration of study (week) | 4 | ≤8 | -0.37 | -0.67, -0.07 | 60.6 |  |
|  |  | 3 | >8 | -0.20 | -0.45, 0.05 | 0.00 |  |
|  | Sample size | 3 | ≤60 participants | -0.03 | -0.42, 0.36 | 0.00 |  |
|  |  | 4 | >60 participants | -0.34 | -0.56, -0.12 | 60.9 |  |
|  | Type of vitamin D | 7 | Vitamin D3 | -0.27 | -0.46, -0.08 | 40.5 |  |
|  |  | - | Vitamin D2 | - | - | - |  |
|  | Baseline levels of 25(OH)D (ng/mL) | 4 | ≥15 ng/mL | -0.34 | -0.56, -0.12 | 60.9 |  |
|  |  | 3 | <15 ng/mL | -0.03 | -0.42, 0.36 | 0.0 |  |
|  | BMI status (kg/m^2^) | 2 | ≥26 | -0.16 | -0.45, 0.14 | 31.7 |  |
|  |  | 3 | ≤25 | -0.03 | -0.42, 0.36 | 0.0 |  |
| ICAM-1 | Type of intervention | 2 | CKD | -51.59 | -148.66, 45.49 | 99.2 | 97.4 |
|  |  | 1 | Other | -0.11 | -0.74, 0.51 | - |  |
|  |  | 2 | Cardiac disease | -0.09 | -0.61, 0.42 | 55.4 |  |
|  | Dosage of vitamin D (IU/day) | 2 | ≤4,000 | -0.09 | -0.61, 0.42 | 55.4 |  |
|  |  | 3 | >4,000 | -10.06 | -15.94, -4.18 | 98.6 |  |
|  | Duration of study (week) | 1 | ≤8 | -0.39 | -0.95, 0.17 | - |  |
|  |  | 4 | >8 | -3.43 | -6.43, -0.42 | 98.1 |  |
|  | Sample size | 3 | ≤60 participants | -0.96 | -2.23, 0.31 | 90.9 |  |
|  |  | 2 | >60 participants | -50.29 | -149.93, 49.35 | 99.2 |  |
|  | Type of vitamin D | 3 | Vitamin D3 | -5.93 | -10.43, -1.42 | 98.4 |  |
|  |  | 2 | Vitamin D2 | -1.14 | -3.70, 1.42 | 96.7 |  |
|  | Baseline levels of 25(OH)D (ng/mL) | 2 | ≥15 ng/mL | -50.55 | -149, 48.56 | 99.2 |  |
|  |  | 3 | <15 ng/mL | -0.77 | -2.14, 0.60 | 93.5 |  |
|  | BMI status (kg/m^2^) | 2 | ≥26 | -50.29 | -149, 49.35 | 99.2 |  |
|  |  | 2 | ≤25 | -1.28 | -3.59, 1.04 | 94.9 |  |
| VCAM-1 | Type of intervention | 3 | CKD | -0.71 | -2.55, 1.13 | 96.5 | 91.2 |
|  |  | 2 | Other | -0.30 | -0.89, 0.29 | 56.6 |  |
|  |  | 2 | Cardiac disease | -0.46 | -1.55, 0.62 | 89.0 |  |
|  | Dosage of vitamin D (IU/day) | 3 | ≤4,000 | -0.49 | -1.11, 0.14 | 79.9 |  |
|  |  | 4 | >4,000 | -0.52 | -1.84, 0.81 | 94.9 |  |
|  | Duration of study (week) | 1 | ≤8 | -1.04 | -1.63, -0.45 | - |  |
|  |  | 6 | >8 | -0.41 | -1.19, 0.36 | 92.0 |  |
|  | Sample size | 3 | ≤60 participants | -1.04 | -2.22, 0.14 | 89.4 |  |
|  |  | 4 | >60 participants | -0.12 | -0.97, 0.73 | 92.0 |  |
|  | Type of vitamin D | 5 | Vitamin D3 | -0.31 | -1.12, 0.49 | 90.9 |  |
|  |  | 2 | Vitamin D2 | -1.03 | -3.23, 1.17 | 95.9 |  |
|  | Baseline levels of 25(OH)D (ng/mL) | 4 | ≥15 ng/mL | -0.40 | -1.39, 0.59 | 92.9 |  |
|  |  | 3 | < 5 ng/mL | -0.65 | -1.89, 0.58 | 92.3 |  |
|  | BMI status (kg/m^2^) | 3 | ≥26 | -0.51 | -1.17, 0.15 | 83.1 |  |
|  |  | 2 | ≤25 | -1.06 | -3.22, 1.11 | 94.6 |  |
| E-selectin | Type of intervention | 2 | CKD | 0.58 | -1.13, 2.30 | 94.2 | 78.8 |
|  |  | 4 | Other | -0.22 | -0.51, 0.07 | 46.5 |  |
|  |  | 4 | Cardiac disease | -0.14 | -0.39, 0.10 | 9.4 |  |
|  | Dosage of vitamin D (IU/day) | 7 | ≤ 4,000 | -0.15 | -0.35, 0.05 | 25.3 |  |
|  |  | 3 | > 4,000 | 0.25 | -0.93, 1.42 | 93.7 |  |
|  | Duration of study (week) | 2 | ≤8 | -0.28 | -0.64, 0.07 | 0.00 |  |
|  |  | 8 | >8 | 0.03 | -0.36, 0.42 | 82.6 |  |
|  | Sample size | 2 | ≤60 participants | -0.40 | -0.82, 0.01 | 0.00 |  |
|  |  | 8 | >60 participants | 0.04 | -0.33, 0.41 | 82.4 |  |
|  | Type of vitamin D | 8 | Vitamin D3 | 0.01 | -0.38, 0.41 | 83.2 |  |
|  |  | 2 | Vitamin D2 | -0.23 | -0.58, 0.11 | 0.0 |  |
|  | Baseline levels of 25(OH)D (ng/mL) | 6 | ≥15 ng/ml | 0.17 | -0.32, 0.67 | 83.9 |  |
|  |  | 4 | <15 ng/ml | -0.35 | -0.57, -0.13 | 40.79 |  |
|  | BMI status (kg/m^2^) | 5 | ≥26 | -0.22 | -0.45, 0.00 | 29.1 |  |
|  |  | 1 | ≤25 | -0.30 | -0.92, 0.32 | - |  |
| Endothelin | Type of intervention | - | CKD | - | - | - | 90.5 |
|  |  | 4 | Other | -0.49 | -1.18, 0.19 | 90.5 |  |
|  |  | - | Cardiac disease | - | - | - |  |
|  | Dosage of vitamin D (IU/day) | 2 | ≤4,000 | -0.91 | -1.22, -0.61 | 0.0 |  |
|  |  | 2 | >4,000 | -0.07 | -1.34, 1.19 | 94.6 |  |
|  | Duration of study (week) | 1 | ≤8 | 0.58 | 0.14, 1.01 | - |  |
|  |  | 3 | >8 | -0.84 | -1.08, -0.60 | 0.00 |  |
|  | Sample size | 1 | ≤60 participants | 0.58 | 0.14, 1.01 | - |  |
|  |  | 3 | >60 participants | -0.84 | -1.08, -0.60 | 0.00 |  |
|  | Type of vitamin D | 3 | Vitamin D3 | -0.84 | -1.08, -0.60 | 0.0 |  |
|  |  | 1 | Vitamin D2 | 0.58 | 0.14, 1.01 | - |  |
|  | Baseline levels of 25(OH)D (ng/mL) | 1 | ≥15 ng/ml | -0.92 | -1.38, -0.47 | - |  |
|  |  | 3 | <15 ng/ml | -0.35 | -1.24, 0.53 | 92.7 |  |
|  | BMI status (kg/m^2^) | 4 | ≥26 | -0.49 | -1.18, 0.19 | 90.5 |  |
|  |  | - | ≤25 | - | - | - |  |

ICAM-1, intercellular adhesion molecule 1; VWF, von willebrand factor; VCAM-1, vascular cell adhesion molecule 1.
