# Supplementary material for: The effects of vitamin D supplementation on endothelial activation among patients with metabolic syndrome and related disorders: a systematic review and meta-analysis of randomized controlled trials
Source: Nutr Metab (Lond). 2018 Nov 29;15:85. doi: 10.1186/s12986-018-0320-9 (PMC6267828; doi:10.1186/s12986-018-0320-9)
Supplement: Supplementary file 3 — The association between vitamin D supplementation and endothelial activation based on sensitivity analysis. (DOCX 13 kb) [file 12986_2018_320_MOESM3_ESM.docx]

**Supplemental file 3.** The association between vitamin D supplementation and endothelial activation based on sensitivity analysis

| Variable | Pre-sensitivity analysis | | | Upper &  lower of  effect size | Post-sensitivity analysis | | |
| --- | --- | --- | --- | --- | --- | --- | --- |
|  | No. of studies included | Pooled SMD  (random effect) | 95% CI |  | Pooled SMD  (random effect) | 95% CI | Excluded  studies |
| VWF | 7 | -0.27 | -0.46, -0.08 | Upper | -0.15 | -0.36, 0.05 | Witham (b) |
|  |  |  |  | Lower | -0.32 | -0.53, -0.12 | Dalan |
| ICAM-1 | 5 | -1.96 | -4.02, 0.09 | Upper | -0.65 | -1.59, 0.27 | Emami Naeini |
|  |  |  |  | Lower | -3.88  -3.42  -3.29 | -7.06, -0.70  -6.4, -0.42  -6.16, -0.43 | Sokol  Arnson  Longenecker |
| VCAM-1 | 7 | -0.50 | -1.19, 0.19 | Upper | -0.24 | -0.88, 0.39 | Assimon |
|  |  |  |  | Lower | -0.75 | -1.34, -0.16 | Zhang |
| E-selectin | 10 | -0.04 | -0.36, 0.28 | Upper | 0.01 | -0.33, 0.35 | Neyestani (b) |
|  |  |  |  | Lower | -0.20 | -0.36, -0.03 | Zhang |
| Endothelin | 4 | -0.49 | -1.18, 0.19 | Upper | -0.35 | -1.23, 0.53 | Neyestani (a) |
|  |  |  |  | Lower | -0.83 | -1.07, -0.59 | Borgi |

VWF, von willebrand factor; ICAM-1, intercellular adhesion molecule 1; VCAM-1, vascular cell adhesion molecule 1
